# The *GATA1s* isoform is normally down-regulated during terminal haematopoietic differentiation and over-expression leads to failure to repress *MYB, CCND2* and *SKI* during erythroid differentiation of K562 cells

**DOI:** 10.1186/1756-8722-5-45

**Published:** 2012-08-01

**Authors:** Christina Halsey, Marie Docherty, Mhairi McNeill, Derek Gilchrist, Michelle Le Brocq, Brenda Gibson, Gerard Graham

**Affiliations:** 1 Institute of Infection, Immunity and Inflammation, Level 3, Glasgow Biomedical Research Centre, 120, University Place, Glasgow, G12 8TA, UK; 2 Department of Paediatric Haematology, Royal Hospital for Sick Children, Glasgow, G3 8SJ, UK

**Keywords:** GATA1 transcription factor, Humans, Down syndrome/*genetics/physiopathology, Leukaemia, Megakaryoblastic, Acute/genetics, Dendritic cells, Cell differentiation/*drug effects

## Abstract

**Background:**

Although *GATA1* is one of the most extensively studied haematopoietic transcription factors little is currently known about the physiological functions of its naturally occurring isoforms *GATA1s* and *GATA1FL* in humans—particularly whether the isoforms have distinct roles in different lineages and whether they have non-redundant roles in haematopoietic differentiation. As well as being of general interest to understanding of haematopoiesis, *GATA1* isoform biology is important for children with Down syndrome associated acute megakaryoblastic leukaemia (DS-AMKL) where *GATA1FL* mutations are an essential driver for disease pathogenesis.

**Methods:**

Human primary cells and cell lines were analyzed using *GATA1* isoform specific PCR. K562 cells expressing *GATA1s* or *GATA1FL* transgenes were used to model the effects of the two isoforms on *in vitro* haematopoietic differentiation.

**Results:**

We found no evidence for lineage specific use of *GATA1* isoforms; however *GATA1s* transcripts, but not *GATA1FL* transcripts, are down-regulated during *in vitro* induction of terminal megakaryocytic and erythroid differentiation in the cell line K562. In addition, transgenic K562-*GATA1s* and K562-*GATA1FL* cells have distinct gene expression profiles both in steady state and during terminal erythroid differentiation, with *GATA1s* expression characterised by lack of repression of *MYB, CCND2* and *SKI*.

**Conclusions:**

These findings support the theory that the *GATA1s* isoform plays a role in the maintenance of proliferative multipotent megakaryocyte-erythroid precursor cells and must be down-regulated prior to terminal differentiation. In addition our data suggest that *SKI* may be a potential therapeutic target for the treatment of children with DS-AMKL.

## Findings

GATA binding protein 1 (*GATA1*, GenBank ID: NM_002049) is a key haematopoietic transcription factor with a pivotal role in differentiation of the erythroid, megakaryocyte, eosinophil and mast cell lineages[1]. The *GATA1* gene produces at least two protein isoforms—the well characterised GATA1 full-length (GATA1FL) isoform and a truncated isoform—GATA1 short (GATA1s). The GATA1FL protein comprises two zinc fingers (which interact with DNA and essential co-factors) a C-terminal tail (of mostly unknown function) and an N-terminal domain (originally thought to confer activation properties to the molecule, but which may also be involved in transcriptional repression [2]). GATA1s lacks the N-terminal domain but is otherwise identical.

The biological role of *GATA1s* in humans is unknown and this isoform received scant attention until the discovery that *GATA1FL* mutations were linked to a highly informative pair of disorders—transient abnormal myelopoiesis (TAM)[3] and acute megakaryoblastic leukaemia (AMKL) [4,5]—seen in children with Down syndrome (constitutional trisomy 21, OMIM ID: 190685). In these disorders the *GATA1FL* mutations are always clustered within the N-terminus, allowing unhindered production of the *GATA1s* isoform. This finding has led to interest in the pathological and physiological role of *GATA1s* in haematopoiesis.

The biological effects of *GATA1s* have been studied using transgenic mice and *in vitro* rescue of *GATA1* deficient cells [6-9]. These papers show that *GATA1s* expression, in the absence of *GATA1FL*, is associated with defects in lineage restriction, alternative cell fate decisions and proliferation/cell cycle control. In particular unopposed *GATA1s* expression is associated with outgrowth of primitive hyper-proliferative megakaryocytic progenitors with a limited capacity to undergo terminal differentiation [6-8]. However, concerns remain about translating results from murine systems into humans since mouse models do not recapitulate all the features of human disease [7,10,11]. In addition, whilst mice produce the two isoforms via alternative translation of a single *GATA1* mRNA [12], humans use alternative splicing of a pre-mRNA to produce both *GATA1FL* and *GATA1s* transcript [13]. Alternative splicing provides a mechanism by which the isoforms could be individually regulated, raising the possibility that alternative isoform usage occurs in particular lineages or at specific stages of haematopoietic differentiation. For these reasons we chose to address three questions regarding the role of *GATA1* isoforms in human haematopoiesis; 1) Do different lineages show different isoform usage? 2) Are isoform expression levels dynamically regulated during haematopoietic differentiation? 3) Do the two isoforms have different effects on the transcription of key target genes during *in vitro* differentiation?

## Results and discussion

### *GATA1* isoforms are not separately expressed in different cell types

**Figure 1:**
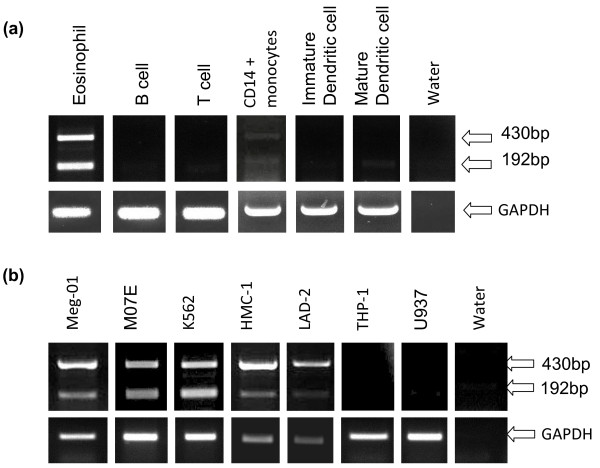
**Expression patterns of *GATA1FL *and *GATA1*s in haematopoietic cells.** (**a**) RT-PCR of human primary cell cDNA using *GATA1* exon 1 and 3 primers (top panel) and *GAPDH* housekeeping gene primers (Bottom panel). Predicted size of *GATA1FL* = 430 bp and *GATA1s* = 192 bp (**b**) RT-PCR of human haematopoietic cell line cDNA using the same primers as (**a**).

Both isoforms are known to be expressed in human CD34^+^ cells [14] and erythroblasts and megakaryocytes [10]. Here we extend these observations by using RT-PCR to detect isoform expression in other human primary cells (Figure 1a). Eosinophils expressed both isoforms whilst, as expected, the lymphoid B and T cells did not express either isoform. We also studied monocytes and dendritic cells since the role of *GATA1* in dendritic cell (DC) maturation is currently debated [15-17]. Human CD14^+^ cell preparations only weakly expressed both isoforms but maturation into DCs was associated with loss of expression of both transcripts both by RT-PCR (Figure 1a) and quantitative PCR (data not shown), arguing against a major role for GATA1 isoforms in DC development.

To overcome concerns about possible contamination of primary preparations with other cell types, a number of cell lines were also studied (Figure 1b). An acute monocytic leukaemia cell line THP-1 and the histiocytic (monocyte/macrophage) cell line U937, did not express either isoform. Expression of both isoforms was seen in the megakaryocytic cell lines Meg-01 and MO7-E along with K562 (a chronic myeloid leukaemia cell line known to be capable of both megakaryocytic and erythroid differentiation *in vitro*). In addition the mast cell lines HMC-1 and LAD2 expressed both isoforms although, interestingly, they appeared to have a relatively weak *GATA1s* band compared to other cell types.

Importantly, in all the cells we examined the two isoforms appear to always be expressed together with no evidence of exclusive *GATA1s* or exclusive *GATA1FL* expression in any of the cell types. This observation argues against a primary role for the two isoforms in lineage specification.

### *GATA1* isoform expression is dynamically regulated during induction of haematopoietic differentiation

**Figure 2:**
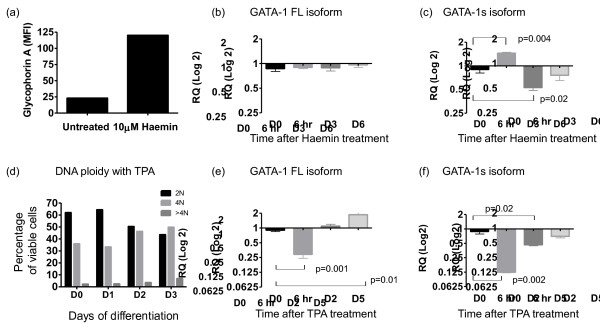
***GATA1s *expression levels during haematopoietic differentiation of K562 cell lines by Haemin and TPA.** (**a**) K562 surface glycophorin A expression measured by single colour FACS after 3 days of Haemin treatment (MFI = −Mean Fluorescence Intensity) (**b**, **c**) quantitative PCR analysis of (**b**) *GATA1FL* and (**c**) *GATA1s,* expression levels during the course of Haemin-induced erythroid differentiation (n = 3), results are expressed as fold change (log2) using day 0 as the calibrator (expression arbitrarily set at 1.0) (**d**) DNA ploidy analysis in K562 cells 0, 1, 2 and 3 days following treatment with the differentiating agent TPA (**e**, **f**) quantitative PCR analysis of (**e**) *GATA1FL* and (**f**) *GATA1s,* expression levels during the course of TPA-induced megakaryocytic differentiation (n = 3), results are expressed as fold change (log2) using day 0 as the calibrator (expression arbitrarily set at 1.0).

It is known that total *GATA1* levels show initial up-regulation during erythroid commitment followed by terminal down-regulation [1,18]. Whether the two *GATA1* isoforms are independently regulated at the transcriptional level during this process is unknown. To investigate this, we used an established *in vitro* model of Haemin-induced erythroid differentiation in K562 cells [19]. Successful differentiation was confirmed by a visible haemoglobinised cell pellet as well as up-regulation of glycophorin A (Figure 2a). Quantitative PCR using *GATA1* isoform specific primers and probes was performed at baseline and then 6 h, day 3 and day 6 following treatment (Figure 2b, c).

*GATA1FL* expression remained relatively constant during differentiation but *GATA1s* showed a significant up-regulation at 6 h and down regulation by day 3 compared to baseline expression levels.

We went on to see whether similar dynamic changes are seen during *in vitro* megakaryocytic differentiation of K562 using TPA [20]. Successful differentiation was confirmed by an increase in DNA ploidy (Figure 2d) and by induction of expression of the megakaryocytic genes *ITGA2B* and *GPB1A* (data not shown). Analysis of *GATA1* isoform expression levels (Figure 2e, f) showed an initial reduction in both isoforms at 6 h. Again, *GATA1s* transcripts remained suppressed at later time-points whilst *GATA1FL* transcripts rose to above baseline.

Although the effects are modest, the fact that levels of the two isoforms can move in opposite directions suggests that their expression can be independently and dynamically regulated. Our observation of *GATA1s* down-regulation from day 3 onwards fits into the prevailing model of *GATA1s* function which suggests that *GATA1s* is important for maintenance of a suitable primed highly proliferative progenitor compartment but that a switch to predominant *GATA1FL* usage may be needed to enact terminal differentiation.

### *GATA1FL* and *GATA1s* transfectants show distinct transcriptional profiles during haemin induced *in vitro* differentiation

**Figure 3:**
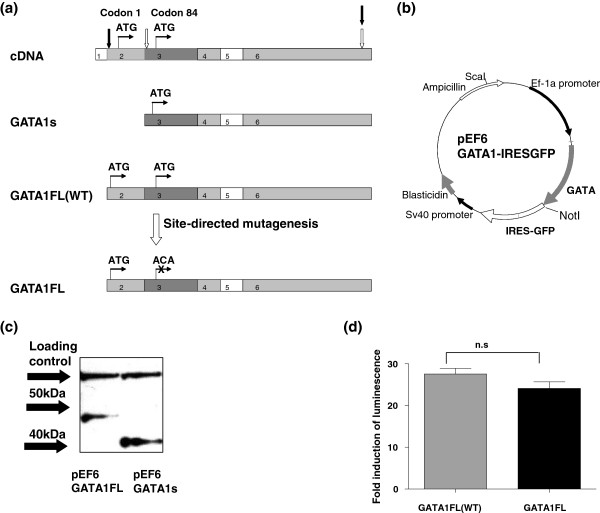
**Production and validation of *GATA1 *isoform specific expression vectors.** (**a**) production of *GATA1s* and *GATA1FL* transcripts from wild type (WT) HEK293 cDNA black arrows represent the position of the *GATA1WT/FL* primers, white arrows mark *GATA1s* primers. (**b**) Map of the pEF6 *GATA1*-IRESGFP expression plasmid. (**c**) Western blot of GATA1 protein expression from *GATA1FL* (lane 1) and *GATA1s* (lane 2) plasmids respectively, using Santa Cruz GATA1 M20 antibody. Arrows show the position of the 40 and 50 kDa size markers, the expected protein sizes are 47 kDa GATA1FL and 40 kDa GATA1s, a loading control is also shown. (**d**) Luciferase assay using a *GATA1* reporter plasmid showing fold induction of luminescence with expression of the WT *GATA1* plasmid versus the mutated *GATA1FL* plasmid (84 Met-Thr).

To extend these observations further we looked at the impact of isoform expression on transcriptional profiles during erythroid differentiation. K562 cells were stably transfected with a *GATA1s*-IRESGFP or *GATA1FL*-IRESGFP expressing plasmid or an empty vector control and high GFP expressers were sorted and used in all subsequent experiments. Details of the construction and validation of these vectors are given in the materials and methods section and outlined in Figure 3. The presence of empty vector had no effect on growth characteristics compared to the parental cell line (p = 0.6, 2-way ANOVA untransfected K562 vs. empty vector). Both *GATA1FL* and *GATA1s* transfection conferred a modest but significant growth advantage to the K562 cells (*GATA1FL vs.* empty vector p = 0.03, *GATA1s vs.* empty vector p = 0.02, student’s 2 tailed *t*-test), but there was no difference between the two isoforms (p = 0.40 *GATA1FL vs. GATA1s*). Since the lower proliferation in the empty vector transfectants could confound comparison of transcriptional profiles it was decided to directly compare *GATA1FL vs.GATA1s* transfectants in subsequent analysis. Although this cannot completely rule out off-target effects related to the presence of the vector the two transfectants should be equally affected by this. We used this system to answer the question of whether the GATA1s and GATA1FL isoforms produce differential effects on transcription when over-expressed in the same cellular context. All experiments were performed in triplicate.

**Figure 4:**
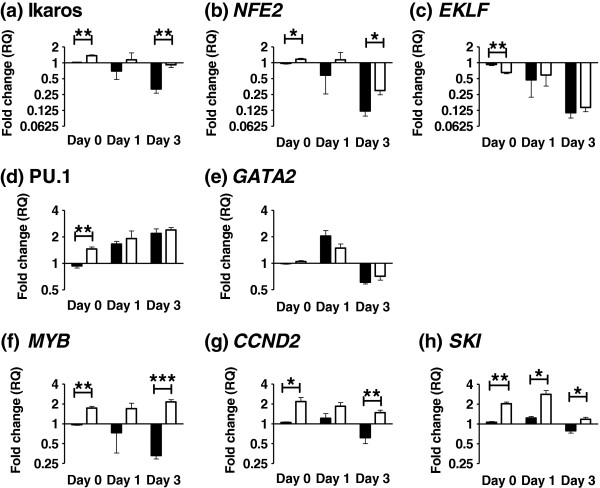
**Gene expression changes in *GATA1 *isoform expressing K562 transgenic cell lines treated with Haemin.** Levels of expression of eight target genes (**a**)-(**h**) in response to Haemin treatment in *GATA1FL-*IRESGFP (black bars) and *GATA1s-*IRESGFP (white bars) transfected K562 cells. Results are expressed as fold change (log2) using day 0 K562 *GATA1FL-*IRESGFP cells as the calibrator (expression arbitrarily set at 1.0). * p ≤ 0.05, ** p ≤ 0.01 and *** p ≤ 0.001.

Using data from microarray studies in Down syndrome AMKL [21,22] and *GATA1* transgenic mice [7] we identified eight potentially informative genes and examined their response to *in vitro* erythroid differentiation in our engineered cell lines. Results of gene expression levels were compared between the *GATA1FL* and *GATA1s* transfectants at day 0 (baseline), day 1 and day 3 of differentiation using ΔΔCT relative quantification (n = 3, Figure 4). *GATA1FL* expressing cells formed a well haemoglobinised cell pellet by day 1 of differentiation whereas *GATA1s* cells formed a haemoglobinsed pellet by day 3 indicating a potential slowing but no absolute block to differentiation.

Two genes—Ikaros and *NFE2*—were down regulated in *GATA1FL* transfectants but to a lesser extent in *GATA1s* transfectants. Inadequate repression of Ikaros (a master regulator of lymphoid differentiation [23]) has already been described in the *GATA1s* transgenic mice [7] and is thought to be consistent with maintenance of a primed multipotent progenitor state. The reduced repression of *NFE2*, an important megakaryocytic transcription factor [24]*,* may partially explain the strong association of *GATA1s* with acute megakaryoblastic leukaemia [4].

A second set of genes—*EKLF,* PU.1 and *GATA2* show some alterations at baseline but no significant differences in response to differentiation. In the *GATA1s* transgenic mouse model [7] PU.1 and *GATA2* showed inadequate repression during *in vitro* megakaryocytic differentiation. The different result seen here may be due to differences between erythroid and megakaryocytic differentiation regimes, expression of a dominant GATA1FL isoform from the endogenous K562 allele or it may reflect differences in the transcriptional response to *GATA1s* between mice and humans.

Perhaps the most interesting genes were those that showed persistent up-regulation in the presence of *GATA1s; MYB*, *CCND2* and *SKI*. *MYB* is a key haematopoietic transcription factor involved in stem cell self-renewal and lineage decisions [25]. In the *GATA1s* transfectants *MYB* persisted at higher levels than undifferentiated *GATA1FL* cells consistent with maintenance of a primitive progenitor state. Interestingly one of the key findings from *GATA1s* transgenic mice is that fetal liver derived megakaryocytic precursors failed to down-regulate *MYB* when differentiated *in vitro*[7]. These results confirm these findings in a completely different (and human) experimental system. Our second gene of interest was *CCND2,* a cell cycle regulator. Since *CCND2* is associated with megakaryocytic differentiation [26] down-regulation would be expected during terminal erythroid differentiation. This is seen with the *GATA1FL* transfectants but not *GATA1s* consistent with its proposed role in bipotent meg-erythroid and megakaryocytic cells. Finally *SKI* is known to show peak expression in murine bipotent meg-erythroid progenitors (MEP) with much lower or undetectable levels in earlier precursors or unipotent megakaryocytic or erythroid precursors [27]. It has been shown to block erythroid differentiation by a direct interaction with GATA1 involving the C-terminal Zinc finger [28]. *SKI* is an important component of the histone deacetylase complex, and over-expression of *SKI* has been implicated in the pathogenesis of acute myeloid leukaemia [29-31]. These observations suggest that a GATA1s-SKI interaction may be important for the maintenance of an expanded MEP compartment with a block to terminal differentiation as seen in transient abnormal myelopoiesis. The precise role of *SKI* in Down syndrome transient abnormal myelopoiesis needs further investigation particularly as this raises the possibility that these children may respond well to relatively non-toxic HDAC inhibitors currently in development [32].

## Conclusions

The data presented here show that the two GATA-1 isoforms can be independently regulated at the transcriptional level during haematopoietic differentiation but we were unable to find any evidence that they are used to specify different lineages. When overexpressed during human erythroid differentiation the two isoforms produce distinct transcriptional profiles which largely mirror those seen in murine models. Finally our observation of sustained *SKI* up-regulation by *GATA1s* suggests a potential target gene which may play an important role in the development of DS-AMKL and is a tractable therapeutic target.

## Methods

### Cell lines

THP-1, U937, K562, and Meg01 cell lines were grown in complete RPMI 1640 with 10% fetal bovine serum (FBS). MO7E were grown in RPMI supplemented with 20% FBS and 10 ng/ml GM-CSF (StemCell Technologies). HMC-1 cells were grown in IMDM supplemented with 10% FBS, 25 mM HEPES, and α-thioglycerol. LAD2 cells were grown in Stem pro-34 SFM media supplemented with stem pro-34 nutrient supplement (Invitrogen) plus 10 ng/ml human Stem Cell Factor (StemCell Technologies).

### Primary cells

Human peripheral blood (PB) eosinophils, T and B cell populations were isolated from human PB as previously described [16]. Cell purities were verified by staining for CD4 (T cells >95%) and CD19 (B cells >95%). Monocytes were isolated using human buffy coats layered over Ficoll-Paque (GE Healthcare). The mononuclear cell fraction was washed and CD14^+^ cells separated using microbeads and an autoMACS Pro separator, following the manufacturer’s protocol (Miltenyi Biotec). Cell purities were consistently >90% by flow cytometry. To generate immature dendritic cells (DCs) (CD14^-^HLA-DR^mid^CD86^mid^) the CD14^+^ cells were grown for 6 days in RPMI 1640, supplemented with HEPES, L-glut (Invitrogen), 5% AB serum, gentamicin (Sigma), 50 ng/mL GM-CSF and 15 ng/mL IL-4 (Miltenyi Biotec). Immunophenotype was confirmed by flow cytometry (MACSQuant, Miltenyi Biotec). Mature DCs (CD14^-^HLA-DR^high^CD83^+^CD86^+^CD80^+^CD25^+^) were generated by resuspension of the immature DC fraction in RPMI 1640 supplemented with 5% AB serum, gentamicin, HEPES, L-glut, 50 ng/mL GM-CSF, 15 ng/mL IL-4, 12.5 μg/mL PolyI:C (Sigma) and 1 μg/mL PGE_2_ (Sigma) for 48 h. Again identity was confirmed by flow cytometry.

### Expression plasmids

*GATA1FL* and *GATA1s* isoforms were amplified from a GATA1-BFT4 expression plasmid using exon 2 (*gttccatggattttcctg*) and exon 3 (*caacagtatggagggaattcc*) primers respectively along with a common exon 6 reverse primer (*gctattctgtgtaccttcaagaac*) (Figure 3a) and cloned into pcDNA3.1 expression plasmids (Invitrogen). The *GATA1FL* plasmid then underwent site directed mutagenesis (QuikChangeII, Stratagene) of the second start codon at position 84 (ATG to ACA), to prevent alternative translation of GATA1s from the same transcript (Figure 3a). Products were subcloned into the pEF6V5hisTOPO expression vector (Invitrogen) and an IRES GFP sequence was ligated into a downstream NotI site to produce the vectors *GATA1s*-IRESGFP, *GATA1FL*-IRESGFP and an Empty vector control containing all vector sequences but no *GATA1* insert: Empty-IRESGFP (Figure 3b). Protein expression from the *GATA1s* and *GATA1FL* vectors was confirmed by western blotting of transfected HEK293 cells (GATA1 M20 antibody, Santa Cruz) (Figure 3c). Transactivation of a *GATA1* luciferase reporter vector was used to confirm that the amino-acid 84 (Met-Thr) change, introduced by site directed mutagenesis, did not produce a functional change in the GATA1FL protein (Figure 3d).

The vectors were stably transfected into K562 cells using Fugene6 (Promega) and maintained in selective Blasticidin containing media. Following several weeks in culture high GFP expressors were isolated using a BD FACSAria cell sorter before use in *in vitro* differentiation experiments.

### Haemin and TPA differentiation

Differentiation of K562 cells was achieved by plating 1 × 10^6^ cells in each well of a 6-well plate and treating for up to 4 days with 100 nM 12-O-tetradecanoyl phorbol-13-acetate (TPA) [33] or 10 μM Haemin (from a 4 mM stock dissolved in NaOH 0.5 M, Tris (pH 7.8))[19]. All experiments were preformed in triplicate.

### Flow cytometry

Cells were washed and treated with human Fc receptor Blocking agent (Miltenyi Biotec) followed by mouse anti-human PE-Cy5 conjugated anti-CD235a (glycophorin A) (BD Pharmingen). Data was acquired from 20,000 gated events on a FACSCalibur instrument (Becton Dickinson) and analyzed using BD CellQuest Pro software.

### DNA ploidy analysis

Cells were washed in cold PBS and added drop-wise to ice-cold ethanol. Following overnight incubation at 4°C cells were spun down and resuspended in 2 ml of Propidium Iodide (PI) mastermix (1xPBS, PI 40 μg/ml, RNase (DNAse-free) 100 μg/ml) and incubated at 37°C for 30 min. Cells were filtered through a 40–70 μm cell strainer and analysed on the flow cytometer as detailed above. A known 2N cell line (Raji) was used as a control and run alongside test samples.

### RT and qPCR

Total RNA was extracted from cell lysates using RNeasy columns (Qiagen) with on-column DNase digestion (RNase-free DNase, Qiagen). RNA (2 μg) was reverse-transcribed using random primers and AffinityScript multiple temperature Reverse Transcriptase (Stratagene). The resulting cDNA was diluted 1:5 and standard RT-PCR was performed using a commercial PCR mastermix (ReddyMix, Abgene) with the following primers: *GATA1* 5′ *ctccgcaaccaccagcccag* 3′ tatggtgagccccctgggatc. The identity of PCR products was confirmed by excision of bands from the agarose gel, purification of DNA using a commercial QIAquick gel extraction method (Qiagen), followed by TOPO cloning into a pCR4TOPO vector (Invitrogen). Plasmid preparations containing insert were then sent for commercial sequencing (http://www.agowa.de).

Quantitative PCR (qPCR) was performed on a 7900HT (Applied Biosystems (ABI)) thermal cycler using Taqman technology (ABI). Previously published *GATA1* isoform primers and probes were used [14]. *GATA1* transcript levels were normalized to *18s rRNA* (one of several reference genes analyzed and found to be unaffected by experimental conditions). Gene expression signatures were analyzed on 384 well microfluidic plates (Taqman low density array (TLDA®plates), ABI) using Universal PCR Mastermix (ABI). Results were analyzed using Sequence Detection Systems (SDS) Software v2.1 and statistical analysis was performed using a two-tailed student’s *t*-test with a p value of ≤0.05 being considered significant.

## Competing interests

The authors declare that they have no competing interests.

## Authors’ contributions

CH, MM, MD and ML performed the experiments, DG, BG, CH and GG helped conceive and design the study. CH drafted the manuscript. All authors read and approved the final manuscript.
